# “Do your homework…and then hope for the best”: the challenges that medical tourism poses to Canadian family physicians’ support of patients’ informed decision-making

**DOI:** 10.1186/1472-6939-14-37

**Published:** 2013-09-22

**Authors:** Jeremy Snyder, Valorie A Crooks, Rory Johnston, Shafik Dharamsi

**Affiliations:** 1 Faculty of Health Sciences, Simon Fraser University, 8888 University Drive, Burnaby, BC, Canada; 2 Department of Geography, Simon Fraser University, 8888 University Drive, Burnaby, BC, Canada; 3 Department of Family Practice, University of British Columbia, David Strangway Building, 3rd Floor 5950 University Boulevard, Vancouver, BC, Canada

## Abstract

**Background:**

Medical tourism—the practice where patients travel internationally to privately access medical care—may limit patients’ regular physicians’ abilities to contribute to the informed decision-making process. We address this issue by examining ways in which Canadian family doctors’ typical involvement in patients’ informed decision-making is challenged when their patients engage in medical tourism.

**Methods:**

Focus groups were held with family physicians practicing in British Columbia, Canada. After receiving ethics approval, letters of invitation were faxed to family physicians in six cities. 22 physicians agreed to participate and focus groups ranged from two to six participants. Questions explored participants’ perceptions of and experiences with medical tourism. A coding scheme was created using inductive and deductive codes that captured issues central to analytic themes identified by the investigators. Extracts of the coded data that dealt with informed decision-making were shared among the investigators in order to identify themes. Four themes were identified, all of which dealt with the challenges that medical tourism poses to family physicians’ abilities to support medical tourists’ informed decision-making. Findings relevant to each theme were contrasted against the existing medical tourism literature so as to assist in understanding their significance.

**Results:**

Four key challenges were identified: 1) confusion and tensions related to the regular domestic physician’s role in decision-making; 2) tendency to shift responsibility related to healthcare outcomes onto the patient because of the regular domestic physician’s reduced role in shared decision-making; 3) strains on the patient-physician relationship and corresponding concern around the responsibility of the foreign physician; and 4) regular domestic physicians’ concerns that treatments sought abroad may not be based on the best available medical evidence on treatment efficacy.

**Conclusions:**

Medical tourism is creating new challenges for Canadian family physicians who now find themselves needing to carefully negotiate their roles and responsibilities in the informed decision-making process of their patients who decide to seek private treatment abroad as medical tourists. These physicians can and should be educated to enable their patients to look critically at the information available about medical tourism providers and to ask critical questions of patients deciding to access care abroad.

## Background

The appropriate role of physicians in aiding patients’ decisions and the informed decision-making process has been the subject of considerable debate. In an era where patient autonomy ranks high in the doctor-patient relationship, paternalistic models – where the physician dictates the patient’s care in what the physician sees as the best interest of the patient – have been widely critiqued [1]. Instead, decision-making models that emphasize the physician’s role in *aiding* patients’ health-related decision-making have gained favour. At one extreme, the physician’s role may be limited to simply providing the patient with information with which the patient may make an informed choice completely on his or her own. In contrast to the paternalistic model, this model has been criticized as being too impersonal and having an unrealistic assumption that patients have clearly articulated values relating to health care [2]. In its place, shared decision-making models have been increasingly championed, including in Canada, where the physician seeks to interpret the patient’s values or helps the patient to choose health-related values by sharing decision-making and reaching a consensus with the patient [3]. Shared health-related decision-making can be understood as “a process of communication in which the physician and patient use unbiased and complete information on the risks and benefits associated with all viable treatment alternatives and information from the patient on personal factors that might make one treatment alternative more preferable than the others to come to a treatment decision” [4]. This model of decision-making is interpersonal, allowing both patient and physician to influence one another during the decision-making process. This process requires trust and attention to the patient’s context, meaning that it is more likely to be successful if the patient and physician have a long-standing and mutually respectful relationship [5].

Medical tourism, the practice where patients travel across national boundaries to access a wide range of private medical care (e.g., necessary and elective surgeries, experimental treatments, dental care, reproductive/fertility services, etc.) in numerous destination nations, has been seen by some scholars as threatening physicians’ abilities to share in and shape the informed decision-making process with their patients [6-8]. In Canada, family physicians serve as gatekeepers within the public system, providing continuing care and monitoring while also determining access to specialist services, and thus serve a crucial role in directing the care of Canadian patients. While patients need not consult with family physicians before accessing private care for elective treatments not covered by the public system, family physicians’ role in the public system is central. Because medical tourists are opting out of their local health care systems, they may not consult with their regular family physician prior to departing, thus missing an opportunity to make their physician aware of the treatment, discuss care options and risks, become informed about how to maintain their continuing medical record, and prepare for postoperative follow-up care upon return [9]. The physician’s involvement in these patients’ informed decision-making can be replaced by foreign providers and medical tourism facilitators, each of whom have a financial interests in encouraging the patient to seek care abroad and thus may not be able to provide the information patients need in order to fully achieve informed consent [6]. For example, in the case of medical tourism facilitators, third parties who arrange for the patient’s care abroad, many do not have medical training and fail to disclose the risks of treatment on their websites. Instead, these websites often include waivers of facilitator liability for any ill effects from treatment received abroad [10,11]. Moreover, the potential brevity of the interaction between the patient and physician or clinic/hospital abroad, coupled with difficulties accessing patient records from outside the patient’s home system, may also complicate shared informed decision-making by the patient and physicians working in medical tourism facilities [6].

In this paper, we present the findings of a thematic analysis of interviews conducted with family physicians in British Columbia, Canada that illustrate the ways in which their patients’ engagement in medical tourism challenges their own involvement in the shared decision-making process. Given the concerns with the influence of the limited amount of third party or ‘neutral’ information available about medical tourism on medical tourists’ decision-making and abilities to provide informed consent [6,7] we consider the impacts of patients’ engagement in medical tourism on the continuing relationship between family physicians in British Columbia and their patients that have gone abroad for care. Medical tourism can sometimes be seen as empowering patients, giving them new care options not available or affordable at home [12]. This is frequently a message of medical tourism guidebooks, which tout medical tourism as a way of shifting authority to patients and away from paternalistic physicians [13]. But, as we will discuss, the global dimension of medical tourism also creates significant barriers to shared informed decision-making between patients and their regular physicians, which can have significant and lasting negative impacts on these patients’ care, welfare, and autonomy and serve in stark contrast to viewing this practice as primarily empowering.

## Methods

The analysis presented in this paper contributes to an exploratory qualitative study designed to examine what Canadian family physicians view their roles and responsibilities to be towards patients in their practice who choose to engage in medical tourism. To address this purpose focus groups were held in the spring of 2011 with family physicians practicing in the province of British Columbia. British Columbia was selected as the provincial site for data collection because it is known that several medical tourism facilitation companies operate there and that patients from the province are indeed opting to travel abroad for private medical care [14]. Six cities that spanned all five of the province’s regional health authorities and varied in size were chosen as locations to run focus groups in so as to capture some degree of diversity in working environment amongst participants.

### Recruitment

Participant recruitment started after approval for this study was received from the Research Ethics Board at Simon Fraser University. After approval was granted we – a team of health service researchers with social science and ethics training - searched the listing of the British Columbia College of Family Physicians directory to identify all family physicians practicing in the six cities selected for data collection. Letters of invitation to participate in the focus group were faxed to all those identified. The letters contained basic information about the study and the focus group time and location and asked that anyone interested in participating call a toll-free line or send an e-mail to reach a study investigator. People receiving these letters were also asked to share details of the study with others in their practice.

In total, 22 family physicians agreed to participate in the study. The focus groups ranged in size from two to six participants. Participants had, on average, been practicing family medicine for 23 years. Twenty of the 22 participants had seen at least one medical tourist in their practices. The total number of medical tourists they had seen ranged significantly, though, from one to 90 (median = 6).

### Data collection

The focus groups were run by two co-moderators and a note-taker was also present at each. All six focus groups lasted between 1.5 and 2 hours. Consistent with the focus group method, the conversations were structured around a series of probes that inquired about a number of topics related to participants’ perceptions of and experiences with medical tourism. The probes were determined following a detailed review of the international medical tourism literature that pertained to decision-making as well as impacts on patients’ home countries so as to establish knowledge gaps and useful areas of inquiry. While the focus group probes guided the conversation, the topics covered in the discussions were very much driven by the participants.

### Analysis

All focus groups were digitally recorded and transcribed verbatim. After transcription was complete the transcripts were uploaded into NVivo in preparation for thematic analysis. Transcripts were independently reviewed by all investigators, after which a face-to-face team meeting was held in order to discuss emerging analytic themes. Following this meeting a coding scheme was created by the second and third authors using inductive and deductive codes that captured issues central to the analytic themes identified by the investigators. The scheme was applied to the transcripts in NVivo by the third author with confirmation on interpretation being sought from the second author.

After coding was finished extracts of the coded data that dealt with issues of informed decision-making, which serves as the focus of the current analysis, were shared among the investigators in order to identify the breadth and depth of themes central to the topic. Four such themes were identified, all of which dealt with the issue of the challenges that medical tourism poses to family physicians’ abilities to support medical tourists’ informed decision-making. The interpretation of these themes was confirmed through review of the raw data independently by the investigators. In keeping with thematic analysis, the findings relevant to each theme were contrasted against the existing medical tourism literature so as to assist in understanding their significance.

## Results

Canadian family physicians encounter patients seeking many different treatment types, including experimental treatments now approved in Canada, treatments for which there are real or perceived waiting times for access, and elective treatments not covered by the public system, among others. Four key challenges to Canadian family physicians’ participation in informed decision-making with patients who engaged in medical tourism were identified through thematic analysis. First, patient travel abroad heightens tensions around the physician’s appropriate role in patient’s health-related decision-making. Second, the global dimension of this practice shifts responsibility for the outcomes of patients’ decisions almost solely onto the patient because of the regular physician’s reduced role in sharing decision-making. Third, medical tourism can put a strain on the relationship between physicians and patients by shifting authority over decision-making to be shared between the patient and physicians abroad, potentially omitting the regular family physician entirely. Finally, Canadian family physicians are challenged in balancing patients’ hope for better health with the best available evidence on treatment efficacy, which is complicated by the fact that patients are pursuing their hopes for effective care abroad where national regulatory regimes may permit forms of care not approved in Canada. These four challenges were each linked by participants to the international nature of medical tourism, where domestic family physicians are less capable of partnering with patients in determining their best course of care abroad. In the remainder of this section we examine the findings relevant to each of these four challenges to family physicians’ involvement in shared, informed decision-making with patients opting for medical tourism. We include verbatim quotations from the focus groups so as to enable the participants to ‘give voice’ to the issues at hand.

### Reshaping the family Physician’s role

The family physicians we spoke with had markedly varied views about what their role should be toward patients’ decision-making around engaging in medical tourism. For some physicians, their role was to help inform and guide patient decision-making but, in the end, to respect these patients’ choices. One physician stated that his duty is to “*give someone the choices*” as “*the practice now is not paternalism where we tell people what to do, but we give them the options and they decide which one to take*”. Similarly, another physician framed this viewpoint in terms of patient autonomy: “*we’re sort of patient partners and so they present something, we just kind of give them our opinion… They’re adults, they’re intelligent, they make their own choices and have…autonomy to do whatever they want to do…so we can just come alongside*”. Both of these responses situate physicians as partners in decision-making rather than as experts.

When a family physician feels that s/he is not able to offer an informed view about medical tourism and thus act as a partner in the decision-making process, the common response to patients is: “*you do your homework …and then hope for the best*”. This stands in contrast to the shared decision-making model that is common in Canadian family medicine practice in that it lessens the physician’s role. For some others, though, it was thought best that physician take a more paternalistic role, protecting patients from harming themselves or being defrauded of their time and money. One physician put it: “a role for a family physician is to protect a patient from that type of care…for us to somehow stop those people from doing that”. This approach is also markedly different from the shared decision-making model that is common in Canadian family medicine practice in that it shifts away from a shared approach. One physician noted that she changed her approach depending on the patient’s personal situation. While she is typically “*delicate*” and “*tip-toey*” with her patients, if the patient has limited financial means and is considering spending a large sum on an unproven, alternative treatment, she will speak more forcefully as there is a “*harm issue here*”.

### Shifting responsibility for decision-making

Participants emphasized that family physicians in British Columbia typically take on a significant role in providing information for patients to use in their informed decision-making process for accessing medical care in the domestic system. In the context of decision-making around international travel to access medical care, however, many participants found their abilities to provide information to be challenged because they felt unprepared to discuss this issue. For example, the physicians we spoke with described the common phenomenon of patients arriving at their offices with stacks of internet print-outs about the procedures and facilities abroad that they were considering accessing: “*I know and it’s very challenging to even understand it ‘cause they often bring you this pile, what do you think of this doctor, what do you think of this centre. And then so you have to kind of wade through that and figure out what’s reasonable*”. Their lack of preparedness for these types of doctor-patient interactions left many wanting to take minimal or no responsibility for engaging in shared informed decision-making around medical tourism.

Because participants generally felt limited in their abilities to share their patients’ medical decision-making by vetting or even simply discussing procedures and facilities abroad, many emphasized that these decisions were the responsibility of the patient and an exercise of the patient’s autonomy. One participant stressed that, given that physicians will typically not be familiar with the facilities and physicians abroad, and sometimes the procedures as well, they will not want to “*take responsibility*” for the patient’s choice: “*So the average physician will go along with the patient and just say well, I really don’t know what it involves but if you think it’s a good idea then you do what you think is best for yourself, and that would be about it*”. This attitude puts the responsibility to find information and to ask critical questions solely on the patient. As one participant explained, “*I don’t hunt [for information], I just say ‘well there could be something and you should look’*”. In other cases, the emphasis on the patient’s choice was framed less in terms of wanting to shift decision-making responsibility onto the patient and more in terms of supporting the patient and ensuring that the patient was aware of the current level of knowledge regarding a particular procedure: “*I would say ‘you know the evidence isn’t really there but I would support you in going to get it, if that's what you think you want to do*”. Whatever the justification, however, there was a clear trend among participants to minimize their involvement in, and thus responsibility over, the decision-making process around seeking private medical care abroad.

In some instances participants spoke to their responsibilities in shared decision-making towards patients traveling abroad for experimental procedures specifically. One participant explained that it was important to tell the patient that the current science does not support the treatment they are seeking, but that “*you have a right to make your own choice”.* The physician’s power to guide the choices of the patient was seen as more limited in the context of seeking experimental care because the gatekeeper function of family physicians is subverted by voluntary and privately funded travel outside of the domestic system. Many of these patients do not approach their physicians to “*ask your permission*”, and the sense is that “*all their plans had been laid and I, what do you say, I’m like ‘good luck … I’ll see you when you get back*”. For this physician, it was important to respect “*…the concept of informed consent…and informed decision-making*” regardless of the type of procedure being sought abroad.

### Straining the doctor-patient relationship

The family physicians we spoke with felt that the international and private nature of medical tourism from Canada could alter the doctor-patient relationship, thus complicating the physician’s role in sharing decision-making. This relationship was felt to be very important to the care of the patient and shared decision-making, where the physician must be able to get to know patients and their needs well over time through establishing multiple forms of continuity. When patients go abroad to receive care from doctors the regular family physician does not know, it can leave this physician disconnected from the patient’s decision-making. In general, participants were concerned that the decision to go abroad for care was indicative of a weakened relationship between the doctor and patient, either because these patients were blaming their doctors for their ill health or because the physician was not facilitating patients’ access to the care they wanted when they wanted it. In the latter case, patients might feel that the physician is “*obstructing their path then that may fracture the relationship*”.

Participants raised many specific ways in which patients’ engagement in medical tourism could strain the doctor-patient relationship and its overall therapeutic potential. In one case, for example, a participant expressed that medical tourism was feeding into a culture of “*instant gratification*” among patients where wait times for procedures did not have to be endured, legal or structural barriers to care access could be circumvented, and there was less need to ensure a close doctor-patient relationship in order to enhance domestic system navigation. Another participant worried that patients who access care abroad that they deem of higher quality than what they can get at home will lose faith in the Canadian system, which “*destroys the relationship*” between doctor and patient. This relationship may also be damaged by the decisions that physicians make on how to provide care for patients after their return home, which is an issue raised by many participants across all the focus groups. One participant noted, for example, that if he chooses not to act on the medical advice of a physician from abroad due to lack of knowledge of the treating physician or the merits of the treatment order, the continuing relationship with the patient may be “*damaged*”. Another participant noted that worries about damaging his relationship with his patients might lead him to giving in to patient demands because “*you don’t want to lose that trust that you’ve built with this patient relationship for so many years*”. Only a few other participants indicated that they may do the same in order to avoid damaging an established relationship.

Though not a common discussion point in the focus groups, it was suggested that the financial implications of privately accessing care abroad put tension on the doctor-patient relationship, making it more difficult to share in decision-making. Intended medical tourists may look to their regular family physicians to provide guidance on cost savings by, for example, seeking feedback on or endorsement of low-cost clinics abroad or requesting letters for insurance companies or government in order to seek reimbursement for this care. One participant spoke of her unwillingness to write a physician’s note for a patient who had participated in medical tourism where this document would state that the patient could not access the same care locally. In this case, the patient needed the note to meet the provincial government’s requirements for reimbursing the patient’s care as the government would not reimburse in cases where treatment is available in the local public system. The physician’s refusal to provide such a note led to that patient changing doctors “*because he doesn’t think I have his best interests at heart”.*

### Balancing hope and evidence

Participants explained that in some cases, patients choose to travel abroad for care because of a serious medical condition that they feel could not be treated in Canada. For some of these patients, there is a tension between maintaining hope that they might be cured or improve their quality of life through seeking care abroad lies and their physician’s concern that this hope is not supported by clinical evidence or that the patient may be exploited. Participants widely recognized the importance of maintaining hope for their patients who want to seek life saving or life changing procedures abroad, saying that they should not simply “*dash their hopes*” and that “*all they have is hope*”. This reality informed their own decisions regarding involvement in patients’ decision-making around seeking care abroad. While participants often expressed concerns about fraudulent experimental care clinics abroad, one noted that “*I’ve seen really good results from some of the other clinics in Germany…yes they [patients] spent a hundred thousand dollars easily and it buys them time and not necessarily a cure but they’re happy with it*”. This was a minority perspective, though, as most participants who discussed the pursuit of experimental care abroad by patients were concerned about the quality of care and high costs and struggled to balance these concerns against not eroding hope in the course of decision-making.

Having hope in itself was stressed as having a positive impact on patients’ quality of life: “*So you may adversely kind of affect your patient in the sense…you’re doing the right thing, but in fact you know they very much need that hope to actually get through the next month*”. On the other hand, most participants made clear that they could not simply endorse a course of care that they did not feel would work should patients consult with them about international care options in the course of decision-making. In these cases, it is important to counsel patients of their concerns because of their continuing responsibility to the patient: “*You have to make them aware that you still have reservations about the effectiveness of the treatment, ‘cause you’re still responsible for that patient”.* Expressing these reservations can be difficult. For example, in such an instance family physicians may not be able to offer recommendations on any effective treatment within Canada, which puts them “*in a very bad place*” in striking a balance between enabling patients to maintain hope while ensuring they meet their own ethical and professional obligations to the patient.

When patients who are driven by the hope of finding a life saving or life changing procedure abroad turn to online sources for information on experimental or alternative treatments it may undermine the physician’s ability to caution patients in the course of decision-making. The concern is that patients will not be able to evaluate the claims being made on websites, which is particularly problematic in cases where patients do not seek advice on these claims from their regular family physicians or where the physician has expressed unwillingness to engage in shared decision-making around medical tourism. One participant described his patient’s point of view as, “*I don’t trust you doctors who are just referring amongst yourselves and here’s someone who’s a healer and you know and they’re doing natural things and that just seems better for me*”. Another noted that if he is suggesting that a patient has a ten percent chance of survival and a physician abroad is suggesting a ninety percent chance of survival, most patients will choose to listen to the advice of these other physicians as they are “*clinging onto hope*” and “*gullible*” and that these realities will weigh heavily on their decision-making processes.

## Discussion

Our focus group discussions identified four challenges to Canadian family physicians’ abilities to partner with their patients in undertaking informed decision-making regarding medical tourism:

1. *Determining the physician’s role in facilitating informed decision-making around medical tourism.* Canadian family physicians are challenged by patients’ involvement in medical tourism in that they must determine to what degree their role is to facilitate, as far as they can, informed decision-making and when dissuading their patients from going abroad is appropriate;

2. *Identifying physician and patient responsibility for finding information of facilities, procedures, and physicians abroad.* For physicians who see their role as partnering in their patients’ informed decision-making around medical tourism, they are challenged in that they may have limited information to give patients, thus shifting responsibility onto these patients;

3. *Maintaining a strong physician-patient relationship in the face of greater patient responsibility for decision-making.* Family physicians are challenged in maintaining a strong and positive relationship with their patients in a context where patients are more empowered to make their own decisions regarding care and ultimately take control over the entire decision-making process; and

4. *Balancing between maintaining patients’ hope for treatment abroad and concerns that these treatments are unproven.* Family physicians are challenged by the tension between supporting their patients’ desire to maintain hope for effective treatment while being wary of complicity in encouraging the pursuit of unproven treatments not offered locally and the impact this tension has on their involvement in patients’ informed decision-making.

In many respects, the four challenges to family physicians in sharing the decision-making of their patients who opt for medical tourism raised in our thematic analysis are not new; instead, they are existing challenges in clinical practice that are heightened by the international dimension of medical tourism. Debates among family physicians regarding their role in patients’ informed decision-making have been taking place for decades, including questions of whether physicians should take a paternalistic, hands off, or partnering role [2,15]. A distinct challenge brought by medical tourism is that family physicians may be less familiar with the patient’s treatment options abroad and therefore less able to serve as a partner in decision-making. Thus, they are often left with two, potentially unpalatable, options: either to leave the information gathering and decision-making almost entirely to the patient or to take a more paternalistic role in attempting to dissuade the patient from going abroad. Physicians encountering this challenge can aid their patients in identifying critical questions to ask of their providers abroad, but this is very different from the partnering role often identified as an ideal in patient decision-making [3,4]. This diminished capacity to participate in patients’ informed decision-making around medical tourism has the effect of shifting responsibility to the patient for making these decisions. As a result, patients have been observed to take advantage of resources from across the globe for information about medical tourism, including facilitator websites, foreign providers, and industry sources [10,11,16,17]. As these international resources are typically tied to the medical tourism industry, responsibility for information sharing during the decision-making process is shifted from family physicians, who would typically be expected to be more neutral information sources, to sources with significant conflicts of interest.

These physicians expressed that maintaining a strong and positive relationship with their patients that results in positive therapeutic outcomes where all parties have meaningful and desired involvement in health-related decision-making is always challenging. The family medicine literature identifies some of these ongoing challenges to be a lack of time to consult with patients, patients and physicians with poor communication skills, and funding structures that do not support consultation [5]. These routine challenges certainly exist in the relationships between Canadian family physicians and their patients who are considering or have opted for medical tourism. Meanwhile, as these same patients become empowered to receive opinions and treatment from outside of Canada, they develop new relationships that hold implications for their regular family doctors and ultimately the doctor-patient relationship. The findings show that these relationships can be with physicians abroad as well as medical tourism facilitators, with both of these groups playing a role in providing information to patients during their decision-making processes. Canadian family physicians, who are used to a gatekeeping role toward their patients, are forced to compete with other providers in having involvement in shared decision-making and ultimately developing and maintaining a relationship, including continuity of care, with these patients (see also [9]). As these foreign providers can market treatments and, for the patient, hopes for better health that are unavailable domestically, Canadian family physicians face new pressures to support the hopes of their patients for treatment, even if they do not feel that these treatments are supported by scientific evidence.

The global nature of medical tourism creates challenges for Canadian family physicians by introducing new partners in patients’ decision-making processes and ultimately whether they are able to become truly informed decision-makers. This is a relatively new phenomenon for many Canadian family physicians, and our conversations revealed wide uncertainty about the appropriate roles of domestic and foreign physicians in supporting informed decision-making and providing care for Canadian patients. While this uncertainty will likely be reduced when and if medical tourism becomes more common, visible, and regulated internationally and within Canada, gaining clarity is challenged by the myriad destination countries involved in this trade – an issue that has been discussed extensively in the medical tourism literature around the legal dimensions of this global practice and the lack of clarity over which jurisdictions hold legal responsibility for what [18]. Just as Canadian family physicians are uncertain as to what role they should take in patient decision-making, medical tourism facilitators and other providers lack norms for their roles [8]. These new stakeholders inhabit very different countries with very different cultures of medical care, further complicating agreement about their appropriate roles in patient decision-making. For example, international patient coordinators in different countries have been found to have varied experiences with and reactions to medical tourists and caregivers that travel with them [19]. While academics have expressed concern that the private funding of medical tourism creates a conflict of interest between patients and providers and compromises the informed decision-making process, such relationships are not uncommon outside of, or even within, Canada [20]. A continued conversation is necessary to help develop at least broad norms and best practices regarding informed patient decision-making and care in the medical tourism sector if these conflicts of interest are to be visible to patients and managed in their interest. Our findings show that Canadian family physicians should be considered a key stakeholder group in such a conversation. This is not to say that these norms can or should be incorporated into the Canadian health care system or family medicine practice, but the globalization of medical care will continue to disrupt the roles of family physicians in their patients’ informed decision-making processes and they should be prepared to address these disruptions.

Canadian family physicians clearly find themselves challenged in supporting the decision-making of their patients. If they wish to become more involved in these decisions, then there is a need to better inform physicians of the risks presented by medical tourism and best practices in reducing these risks. Such information is not likely to take the form of recommending for or against specific countries, facilities, or physicians abroad given the large number and diversity of medical tourism providers and the lack of reliable measures of provider quality. Rather, physicians can and should be educated to enable their patients to look critically at the information available about medical tourism providers and to ask critical questions before deciding whether to access care abroad [21-23]. While this form of physician involvement might be seen on the one hand as paternalistic, attempting to dissuade patients from becoming medical tourists, the critical nature of these questions can be aimed at empowering patients to be better advocates for their own health. On the other hand, physician participation in this decision-making might be seen as an endorsement of medical tourism by their patients, which raises distinct concerns around liability and the limits of these physicians’ professional responsibilities [24]. While family physicians may wish to be careful in seeming to endorse a practice with which they are unfamiliar or uncomfortable, partnering with patients in looking critically at their decision-making regarding medical tourism and whether it is truly informed allows physicians to maintain their relationships with their patients and not relegate all decision-making responsibility to patients and third party providers.

In this paper we have focused on the perspective of family physicians and the challenges that they face in sharing or not sharing in the informed decision-making of their patients who travel abroad for care as medical tourists. As such, this discussion does not represent the challenges and concerns of destination country physicians. Just as Canadians family physicians are challenged by the global nature of medical tourism and pluralism around cultures of care, destination country physicians are likely to face these challenges as well. Should a destination country physician consult with and treat patients from a diverse range of countries and cultures, they will likely find it extremely difficult to cope with and support different expectations around information sharing and decision-making. As we have already stated, our findings point to a need to inform Canadian family physicians about medical tourism, including the risks it poses to patients and the means of managing these risks. A similar education effort is needed for destination country providers in order for them to serve as effective partners with their patients from abroad. Other, unique challenges are likely faced by these providers, and therefore there is also great need for research into the perspectives of destination country physicians who serve international patients.

The discussion in this article has focused on the Canada as a country from which patients travel abroad for private health care via the medical tourism industry. Our Canadian focus may limit the transferability of the findings presented here to other home countries for medical tourists. In particular, the public nature of health funding in Canada means that these results may not carry over to contexts with different funding systems, such as the United States [8]. Moreover, Canadian family physicians have a strong gatekeeping role that is not found in all primary care tiers, meaning that Canadian family physicians may be accustomed to a greater amount of control over patients’ access to specialist care and surgeries and the decision-making that surrounds this access than exists in health systems that offer patients more direct access to these same types of care [24]. That said, all physicians who treat patients considering engaging in medical tourism will be faced with challenges regarding how to define and operationalize their roles towards these patients. The global nature of medical tourism creates new opportunities for patients, which can be empowering and beneficial for these individuals. At the same time, the introduction of new and potentially competing care providers may challenge the relationship between all physicians and patients along with the decision-making process and ultimately create pressure to match the treatments and hope offered by facilities abroad. We expect, then, that these findings will be applicable in other settings despite differing health system, health care, and health service contexts, though the relevance of each challenge will we have identified here will need to be tailored to these differing contexts. We thus believe there is a strong rationale for undertaking similar research in other countries so as to determine the role that context plays in shaping the challenges that patients’ regular physicians are experiencing in light of patients’ involvement in medical tourism.

### Limitations

There are two main limitations of this study. First, no data exist that enable us to know how representative the participants are of all family physicians in British Columbia in relation to how many medical tourists they have seen in their practice. While this is a qualitative study and has thus not sought to be generalizable, it would have been very useful to have a population-level sense as to how many medical tourists family physicians are typically seeing in their practices each year across the province. Second, there are limitations inherent to the focus group method that are relevant to this study. For example, some participants talked more than others and in one case a particularly outspoken participant monopolized a short part of the discussion. While we did our best to moderate such behaviours and to encourage conversation among all participants, they nonetheless did occur.

## Conclusions

The growing popularity of medical tourism poses significant disruptions to health systems that have evolved to manage behaviours of care providers and health system users within a relatively closed domestic context. The role of Canadian family physicians in health-related decision-making is one area that has been demonstrated to have been unsettled when the care their patients are seeking is outside of the country. To examine this we held a series of focus groups with family physicians in the province of British Columbia. The experiences and outlooks of family physicians shared during these focus groups suggest that: family physicians are uncertain of their degree of engagement with information sharing around medical tourism decision-making; medical tourists resultantly shoulder a greater burden of health-related decision-making than patients who pursue care within their home health system; the relationships between family physicians and their patients can become strained when patients’ expectations for their family physician’s role in accessing care abroad are mismatched with their physician’s own knowledge and professional norms; and family physicians often strike a balance between their awareness of the role that ‘hope from care abroad’ can play in sustaining their patients’ physical and emotional well-being with their concerns for the real financial and physical harms that certain courses of unproven treatment available in loosely regulated contexts can inflict on patients and their families. Most family physicians we have talked to have adopted a hands off approach toward their patients that have traveled abroad for care out of the uncertainty that this practice brings with it. Thus, medical colleges and educators can benefit family physicians and patients alike with the creation of clear professional norms and standards that specifically address the appropriate role of family physicians in their patients’ informed decision-making process when the care they seek is privately delivered outside of the country.

## Competing interests

The authors declare that they have no competing interests.

## Authors’ contributions

JS participated in three focus groups and wrote the background, results, and discussion sections of the manuscript. VAC wrote the grant that funded this research, participated in 4 focus groups, wrote the methods section, and edited the full manuscript. RJ participated in 6 focus groups, wrote the conclusion, and edited the full manuscript. SD participated in 3 focus groups, wrote the abstract, and edited the full manuscript. All authors contributed to the analysis of the focus group transcripts. All authors read and approved the final manuscript.

## Pre-publication history

The pre-publication history for this paper can be accessed here:

http://www.biomedcentral.com/1472-6939/14/37/prepub
